# Eye Tracking in Virtual Reality

**DOI:** 10.16910/jemr.12.1.3

**Published:** 2019-04-05

**Authors:** Viviane Clay, Peter König, Sabine König

**Affiliations:** University of Osnabrück, Germany; University Medical Center Hamburg, Eppendorf, Germany

**Keywords:** Eye movement, eye tracking, virtual reality, VR, smooth pursuit, region of interest, gaze

## Abstract

The intent of this paper is to provide an introduction into the bourgeoning field of eye tracking in Virtual Reality (VR). VR itself is an emerging technology on the consumer market, which will create many new opportunities in research. It offers a lab environment with high immersion and close alignment with reality. An experiment which is using VR takes place in a highly controlled environment and allows for a more in-depth amount of information to be gathered about the actions of a subject. Techniques for eye tracking were introduced more than a century ago and are now an established technique in psychological experiments, yet recent development makes it versatile and affordable. In combination, these two techniques allow unprecedented monitoring and control of human behavior in semi-realistic conditions. This paper will explore the methods and tools which can be applied in the implementation of experiments using eye tracking in VR following the example of one case study. Accompanying the technical descriptions, we present research that displays the effectiveness of the technology and show what kind of results can be obtained when using eye tracking in VR. It is meant to guide the reader through the process of bringing VR in combination with eye tracking into the lab and to inspire ideas for new experiments.

## Introduction

Recent technical advances have led to a rapid development of Virtual Reality (VR) technologies. Development of VR is partly driven by the gaming industry, which produces VR kits in large volume for commercial purposes. Thus, it is continuously further developed and improved, leading to higher resolutions, higher refresh rates and larger fields of view. High performance displays, graphics cards and other computer hardware make it possible to produce powerful Virtual Reality kits at a reasonable price (1). These developments turn Virtual Reality into a highly valuable and more accessible research tool.

Virtual Reality has the advantage of a well-controlled experiment setup while still giving the subject freedom of movement and placing it in a relatively natural environment. It is possible for the subject to look in all directions by moving the head, just like in the real world. Simultaneous to head movement, the stimuli placement in relation to the subject’s position can be measured with high precision. In fact, whole body movements such as turning towards objects or even walking can be implemented. Through the synchronization of body movements and the images provided to the eyes, high immersion of the subject with the virtual environment can be reached. By providing the senses with information about this environment, subjects gain a sense of presence in the non-physical environment (2). This facilitates a more natural interaction with artificially created stimuli. Motion tracking allows for the recording of all movements made by the subjects. Thus, behavior is tracked under controlled conditions that can be identical over multiple trials. The more intuitive exploration of a 3D world by the subject and the correspondence between subject movements and changes in the environment increase ecologic validity of the experimental paradigms. Thus, the development of VR is in line with the efforts of conducting experiments under less artificial conditions, deemed necessary to truly understand cognitive processes (3).

Eye-tracking is a well-established technique and widely used to investigate human cognition. It was first used at the beginning of the 20th century by using specific contact lenses with a pointer attached to them (4). 30 years later, this technique was optimized by using light beams and recording their reflection on film (5). Modern approaches to eye movement research developed during the 1960’s (6, 7) and have been further refined ever since. The exact means of how to monitor eye movements has changed considerably over the past years. Today, video-based systems using computer vision techniques are dominant (8). Due to the development of small, high quality cameras for devices like smart phones, it is now possible to have light and convenient eye tracking systems that can even fit into a VR headset or portable glasses. These allow for fast and accurate monitoring of eye movements, delivering a considerable amount of data. Due to the close relation between eye movements and cognition (9, 10), eye tracking has received increased attention in a wide variety of experimental setups. With the technical advances and the increasing amount of research in the field of eye tracking, it has now advanced to a technology that can be fruitfully used in a wide variety of setups to investigate human cognitive processes.

In comparison to classical eye tracking, eye tracking in Virtual Reality (VR) is a relatively new and a promising development with its first appearance in the literature at the beginning of this century ( 11). It opens numerous new possibilities for conducting research concerning human perception and behavior. It provides researchers with tools that were previously unavailable. These tools include full body motion tracking by the VR system as well as gaze tracking by the eye tracker. While the subject finds itself in a relatively natural environment that reacts to its movements and actions, all experimental settings can be controlled specifically. The combination of eye tracking and VR makes it possible to calculate the gaze of the subject in 3D space and observe where the subject is looking during the session. Contrary to real world eye tracking, it is easy in VR eye tracking to define regions of interest in 3D space and trace the points in time to determine when the regions were looked at. The combined technique of eye tracking and VR with the advantages of more natural stimuli, more natural movement, controlled environment and controlled data collection makes it possible to answer many research questions in a radically innovative way.

In this paper, an introduction will be provided into technical and practical aspects of eye tracking in VR with the motivation to make it more widespread and easily accessible. A brief overview will be given of this new combination of methods as well as a detailed description of how to set it up and implement it in your own lab. We will examine this tool's usage scenarios in research environments, as well as its advantages and potential shortcomings. Also, we will describe what types of data can be collected. The emphasis is put on the use of VR systems with a motion-tracker and an inbuilt eye-tracker since these are the primary methods of collecting data. There are multiple software and hardware solutions out there that can be used. No statement is intended concerning the best soft- or hardware to use, partially because our experience is biased by the hardware and software that we use and partially because it is a booming industry with new options appearing on a regular basis. Tools and software will be made available by appropriate means. Some technical aspects about the implementation might have changed or become easier by the time this paper is read. The aim is to give a basic introduction and a possible guideline for people new to this area. This information should be helpful to get a good start and our sample data can show anyone considering research with this technique the vast possibilities of VR and inspire new ideas for future experiments.

## Methods

### Components of an Experimental Setup

A complete experimental setup using eye tracking in VR contains many parts. Here, an overview is given of hardware and software components of typical setups.

**Figure 1.**
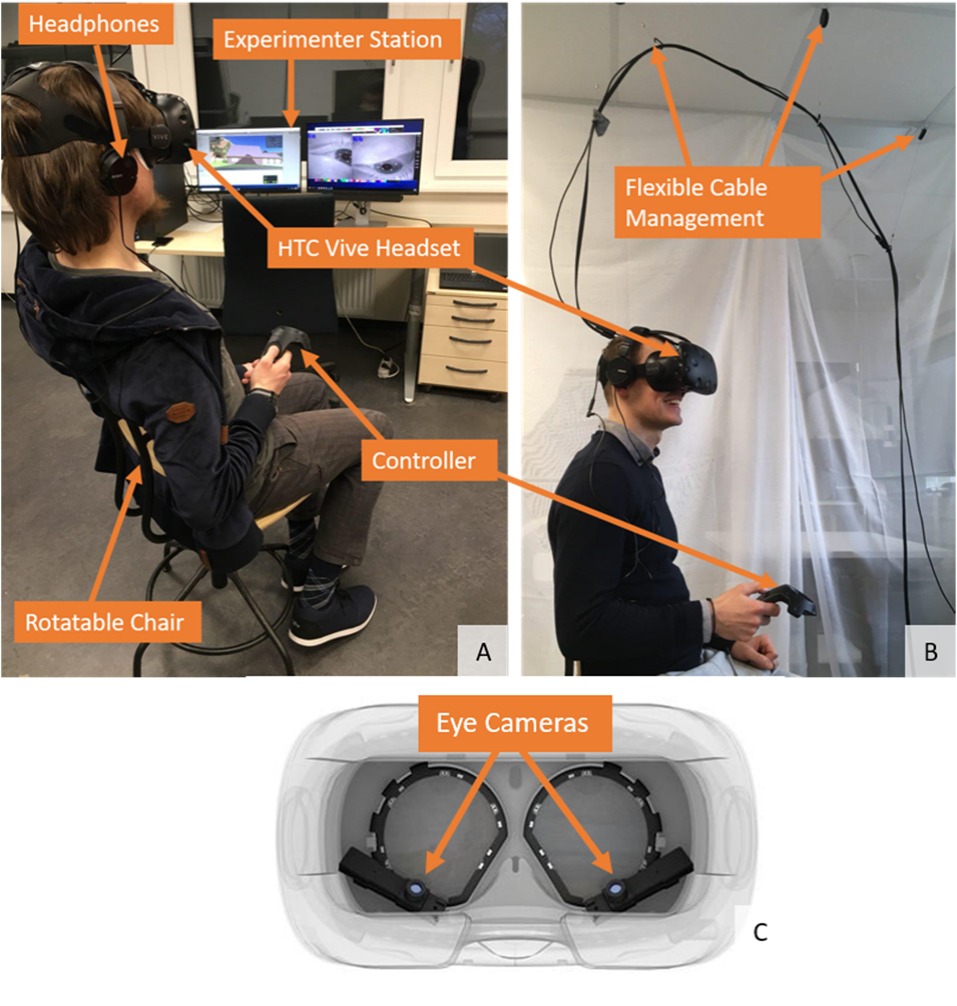
Our HTC Vive setup. (A) Participant sitting in a swivel chair during a session. Walking in the virtual world is done with the controller in our experiment. (B) The flexible cable management system keeps the cables from tangling up and getting in the way of the subject during the session. (C) Pupil Labs Eye-Tracker inside the HTC Vive Headset. Image source: https://pupil-labs.com/vr-ar/ (21.10.2017)

**VR-Hardware set.** This includes a Head-mounted display (HMD), motion trackers and if needed controllers for interaction with the virtual world (Figure 1A). A full high-quality set with these components is offered by Oculus^1^ and the Valve Corporation together with HTC^2^. Another alternative is the PlayStation VR by Sony^3^ for which you can also develop applications with Unity (12). Microsoft also offers consumer virtual reality headsets for which you can develop your own applications^4^. Simple and cheap solutions using only a smartphone screen and a cardboard box are available as well^5^. These are however not optimal for research due to a lower refresh rate, less computing power and no existing eye tracking solutions so far^6^.

**Software.** Common tools to design virtual experiments are Unity^7^, the Unreal Engine^8^ or Vizard^9^. The first two are game engines and are available for free while the latter is more specific for VR. Unity uses JavaScript and C# as programming languages. Unreal uses C++ and possesses a visual scripting system. In Vizard, scripting is done with Python. With all three of these you can create simple or elaborate 3D environments, script different object behaviors and extract information about the subject’s behavior.

**PC hardware.** In spite of the dramatic development of PC hardware in the last three decades, current VR applications test computing power even of the latest systems. This especially applies when rendering a complex scene with many, possibly dynamic objects, detailed textures or changes in lighting. It is important to run the VR environment with a high frame rate, since response lags and jittery movements easily lead to motion sickness in test subjects. The computer typically serves as an interface between the eye-tracker and the VR hardware as well as for collecting all data about the subject’s behavior.

**Eye-tracker.** These are specifically made for different types of head-sets. Companies, which currently offer Eye-trackers for HMDs, are Pupil Labs (Figure 1C)^10^ and Tobii^11^. FOVE currently offers an HMD with an already integrated eye tracker^12^.

**Headphones**. These reduce distracting sounds from outside and support a strong immersion. They can of course also be used for specific auditory experiments. Sounds can be played from different sources in 3D space and can thereby vary in volume depending how the subject turns its head (Figure 1A).

**Cable management.** Most VR setups involve multiple cables connecting the HMD (and the eye tracker) to the computer. As subjects usually move in VR settings, they might get tangled up in the cables from the headset. This can be avoided by installing a simple cable management system (Figure 1B), which we therefore highly recommend. Some vendors offer wireless solutions for HMDs^13^ which can be a good alternative. However, most HMD eye trackers will still require some sort of cable management.

**Disposable hygiene covers.** VR setups are made with single customers in mind. However, in a lab environment many people will use the same headset. Specifically, the headset cushion will touch the subject’s face and the warmth induces sweat. Thus, to have a comfortable experience, and to increase sanitation procedures, disposable covers where the headset would touch the subjects’ skin are highly recommended.

### Motion Sickness, Fatigue and Other Problems in VR

Motion sickness is usually caused by a disparity between visual and vestibular stimuli. For example, inside of a boat, motion sickness is caused because the eyes see a stable scene, but the vestibular system detects movement. The opposite effect occurs in VR. In VR, the eyes detect movement of the scene and apparent movement of the player while the actual person is in most cases sitting stationary on a chair. This difference between the input from the eyes and the input from the vestibular system causes the main part of the motion sickness in VR. Maintaining features essential to the experiment, such as being able to walk around or using a bigger, more complex scene is difficult.

The simplest solution to avoid motion sickness is to design a virtual environment, where the subject doesn’t have to walk. The most common way of getting around in gaming VR is teleportation^14^ since this can be implemented in a manner that causes only minor motion sickness due to the reduced sensory mismatch. However, this is not a very natural way of movement, eliminating this option for many research questions. An alternative could be to let the subject walk on a treadmill in the real world so that the perceived movements match the movement seen in VR. However, this is difficult to implement, especially when the subject can move freely in all directions. A solution to that can be to use an omnidirectional treadmill, but this technology is not very well developed yet and quite expensive. As an alternative it is possible to let the subject freely rotate sitting on a swivel chair to omit translational movements in real space. Overall, the best decision concerning movement in VR depends on one’s experiment and the type of question that one would like to answer. In addition, there is currently a substantial amount of development in this field and there are new ideas for preventing motion sickness coming up frequently.

Besides optimizing the required movement in VR, tweaking the details of the 3D environment and experimental settings helps to minimize motion sickness. Subjects in VR are much more sensitive to little disruptions than when looking at a conventional monitor because they take on a higher expectation of reality. While for example computer games may have latencies of 50ms or higher, in VR a latency rate of more than 15-20ms can already cause severe dizziness and nausea (13). This is because a subject in VR assumes that the virtual world follows the same rules as the real world. So if the subject turns its head and there is even a slight delay of the virtual world turning with it, it is noted as a disruption and causes sickness. Besides the latency, a varying frame rate can cause people to experience nausea as well. As a VR experiment developer, one should therefore keep the framerate in mind when deciding on the complexity of the experiment scene.

Besides motion sickness, it can also be very exhausting for most subjects to keep the headset on for an extended amount of time. Since it is a bit heavy, it is pulling the head of the subject forwards and some complaints have been made about neck pain or pressure on the nose. Also, on the warmer summer days, it can become very hot under the headset and makes most subjects sweat. To keep the subjects comfortable sufficient air conditioning should be made available.

**Figure 2.**
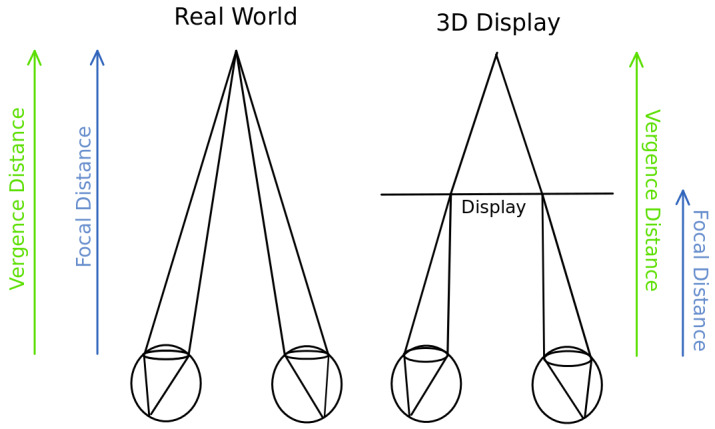
(left) real world: vergence = focal distance. (right) Focal distance always stays the same in VR.

Another challenge of VR is the disparity between vergence and focus (Figure 2). This is called focus-accommodation-conflict (14). In the real world our brain receives depth information from vergence and from the focus of the lens of the eyes. In contrast, we only receive information about vergence in VR. Since the virtual scene is only presented on a single plane at fixed distance, namely the HMD display, one cannot extract any depth information out of the focus of the lens. For most people, this is not a big problem even though some people cannot extract much depth information from vergence alone and have a weaker 3D experience because of that. It can also cause eye-strain and fatigue (15). The additional lack of focus blur can lead to a different perception of size and distance of objects in the virtual environment ( 16). Thus, present technology may still limit the usability of VR in terms of motion sickness and fatigue. However, there is much work going into improving those shortcomings. Solutions such as eye tracking based foveated rendering including focus blur have already been proposed ( 17, 18).

### Eye Tracker Calibration and Validation in VR

Calibration and validation are common practices to ensure accuracy when performing eye tracking measurements. For both procedures, the subject is shown target points in different locations on the screen and is instructed to fixate on them. During the calibration, the eye-tracker uses these targets as reference points to adjust its calculation of the gaze to match the location where the subject is looking. During validation, the visual angle between the coordinates returned by the eye-tracker and the actual coordinates of the target are calculated and provide a quality measure of the calibration. In our experience, the precision of eye tracking slowly deteriorates due to drifts, e.g., slight slips of the headset on the subject’s head during the experiment in VR. Therefore, it makes sense to repeat these two procedures every five to ten minutes during the experiment. There are methods for continuous recalibration available (19). However, they use additional knowledge about the subjects’ viewing behaviors e.g. due to highly salient stimuli, movement in the scene or due to performing mouse clicks. Therefore, they interfere with the experimental setup and are limited to special situations. Thus, repeated calibrations are often needed during VR experiments to counteract possible slippage of the headset and ensure exact eye tracking data.

The important part when implementing a calibration or validation procedure is to show the fixation points in screen space and not in world space. This makes them move together with the head of the subject so that it isn’t possible to turn the head towards the targets in the periphery in order to map these to the center of the visual field. This ensures that all the targets are shown at the intended positions and that the whole visual field is covered. Furthermore, it makes sense to validate mainly in the center of the visual field since the peripheral parts have a lower effective resolution in VR (20). This contributes to the subject turning its head towards the object he wants to look at in the VR environment instead of moving the eyes to far eccentricities. Due to that it makes sense to ensure higher accuracy in the foveal parts of the visual field.

### Combining Eye Tracking and VR

To find out where the subject is looking in a 3D world, one needs to calculate the 3D gaze vector going from the subjects’ eyes into the direction it is looking. When using a 3D eye model for pupil detection, the eye-tracker can already determine a 3D gaze. Depth can theoretically be calculated from divergence of the two eyes by calculating the crossing point of the gaze from both eyes. However, this calculation is imprecise and only returns acceptable results for the most perfect calibrations (21). For example, when trying to differentiate a gaze looking at an object of 20m distance compared to infinite distance based on gaze divergence and assuming an interocular distance of 70mm, one would have to make this judgment by using a very small difference in degree of divergence (Cos(α) = 0.07m / 20m = 0.0035. α ~ 0.2°). Thus, the estimation of relative depth at distances large compared to inter ocular distance require a degree of precision which is infeasible. Since this amount of precision is hard to keep up with the present precision of the eye-tracking technology, we must use a more reliable approach for depth calculation. It has been shown that using multiple features to regress the gaze depth can lead to improved results (22). Here, we propose a much simpler approach, which makes use of the fact that in the virtual world all exact object locations are known and the gaze depth typically stops where a surface is reached.

In VR, we have the advantage of having a 3D eye model and the complete knowledge of the distance between eyes and objects available. This makes it possible to simply calculate the depth of the gaze point in 3D space. Assuming that the spatial extent of the fixated object is large compared to the inaccuracies of the eye tracker, this delivers good results. To combine the eye tracking data with the VR environment, we need to convert the 2D gaze location into a 3D vector in the virtual world. For this approach, we start out with the 2D normalized eye-position of both eyes. This 2D position can then be converted into a 3D gaze into the virtual world based on the head position and rotation in the world. From this 3D gaze, one can then retrieve the depth information by calculating the next intersection with an object in the virtual world. Calculating the 3D gaze vector is relatively easy inside of the game engine. It can be done either for each eye individually or using the cyclopean eye position. In this case, we use the latter by calculating the average of both eyes.

**Figure 3.**
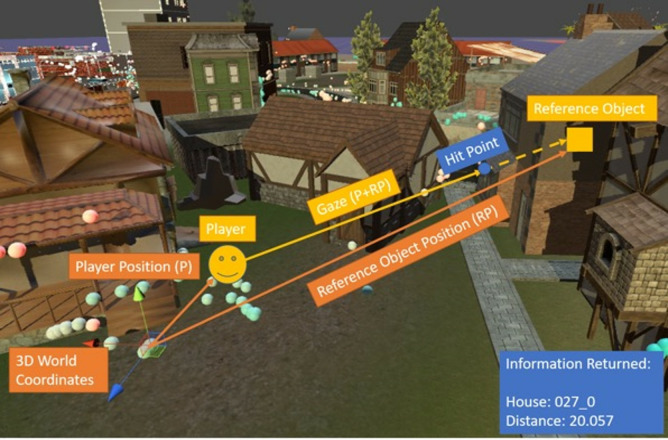
Ray cast (yellow) inside of Unity going from the player to the reference object. The first intersection with an object is taken as the hit point. Colored spheres visualize previous hit points of the players gaze.

Next, one needs to calculate the gaze vector from the cyclopean horizontal and vertical coordinates of the subject’s eyes. This must be done with respect to the subject’s position and head orientation. Since in VR the subject’s head can rotate along three axes and move its position within a certain radius as well, it would be hard to calculate this by hand. An easy solution for this problem is to create a child object^15^ of the subject. The position of this object will vary within a coordinate system relative to the subject’s head. This reference object will be moved within its sub-coordinate system according to the movement of the eyes and the movement of the head. This means that if the player looks to the right the object moves to the right in respect to the position and rotation of the subject’s head. When now drawing a vector from the head position through the position of this reference object inside of the virtual world’s coordinate system, the vector represents the gaze in 3D (see figure 3). This vector is then the subject’s gaze vector and can also be normalized for simplicity. The gaze vector can then simply be obtained by subtracting the head’s position from the position of our reference point. With this procedure, one can turn 2D gaze coordinates into a 3D gaze vector inside the game engine.

### Define Regions of Interest

After calculating a gaze vector, the next step is to determine whether the subject is looking at a specific object or region of interest. So far, the gaze vector only provides a direction. To calculate the distance from the eye to the object looked at, one needs to determine the intersection between this vector and the objects in the virtual world. For this purpose, one can make use of the ray cast system present in game development software like Unity or the Unreal Engine. The way it works can be imagined like shooting an invisible ray from an origin into one direction. This ray then detects when it hits an object and returns information about the hit point and the object that it hit. In our case, we would shoot a ray from the subject’s head position into the direction of our gaze vector. From this ray, one can then extract information about the first object that it intersects with.

An object that should be detectable by a ray needs to have a collider attached to it. In this way, one can define regions of interest (ROI). The size and shape of the collider determines also the size and shape of the ROI. Whenever the ray intersects with the collider around a ROI, we extract the name of the object that is attached to the collider and the length of the ray up to the hit point (distance of the eye of the player to the object). If it proves necessary to have colliders in the 3D environment that do not mark ROIs but are just there for physics purposes and should not be recorded with the help of ray casting, one can use tags to distinguish the two kinds of colliders. For example, it is possible to create a tag for every ROI and then only record intersections of the gaze vector with tagged colliders. The information extracted from the ray cast can then be saved with the same frame rate used for the gaze vector recordings.

Extracting the intersection of the gaze vector and the first hit point requires computational power that might slow down the VR application. Therefore, we would recommend doing this offline after the session to avoid slowing down the VR application with unnecessary computations. At this point, it makes sense to already sort out those data points where the eye tracker gives a low confidence. These are either data points when the subject had its eyes closed or when the eye tracker had problems identifying the pupil. As long as the precision of the eye tracker is sufficient to differentiate fixations on typical objects at different depths, this method produces reliable depth data for the length of the gaze vector.

### Some Points of Caution

Different software often uses varying coordinate systems. Combining data from an eye tracking software with data from a 3D Engine and analyzing this data with yet another software makes it important to watch out for these formal differences. Often x, y and z do not refer to identical dimensions between programs. This also needs to be considered, when analyzing data off-line. Another conversion issue is that the point of origin on a 2D plane can be in a corner or in the middle of the plane. For instance, Pupil Labs uses a normalized coordinate system with the origin 0,0 at the bottom left and 1,1 at the top right, while Unity has the origin at a plane’s center. For valid results, it is necessary to carefully treat the reference frames at different steps and be explicit in the documentation for later users.

Another problem is the difference in frame rates between the different applications. The game engine, which is running a VR application, usually has a lower frame rate than the eye-tracker. The frame rate in a 3D application can also vary depending on the object that is in sight. It can suddenly drop when looking at a very complex object and skyrocket when looking at a simple object. Solutions to increase and stabilize frame rates such as like foveated rendering (23) are under development and expected to be accessible soon. With a large, immersive 3D environment, a drop of the frame rate is difficult to avoid, even with powerful hardware. It should be considered when planning to obtain detailed heat maps or other information that require a high eye-tracking frame rate. In general, it is extremely hard to collect data with a consistently high frame. If this is needed you should design a very simple, low-poly 3D scene without light changes or other effects to reduce the computational complexity and thus ensure recording at a high frame rate. Pupil Labs provides timestamps to record eye movements with a high frame rate externally. This enables you to later synchronize them with the user’s movements in the VR environment. However, it is desirable to keep the frame rate in your application high for later synchronization with the high frequency pupil recordings. Otherwise, the precision of the 3D gaze vector will deteriorate due to bad synchronization and frame interpolation. If it is sufficient for the question addressed, recording gaze vectors at a low frame rate can be a solution to guaranteeing stable and complete recordings. However, if one is interested in precisely defined fixations or saccade dynamics, it is necessary to address the problem of synchronization and higher sampling rate of the eye tracking. In general, one should always aim for a high and relatively constant frame rate to make the synchronized between the eye tracker and the VR engine easier and more accurate.

In VR fixations are not as well defined as during eye tracking when looking at a static 2D scene. A clear way to differentiate fixations from smooth pursuit or optokinetic nystagmus needs to be established. It has to be kept in mind that for eye tracking in a 3D environment established concepts like fixations and saccades are not as clearly defined as in front of a 2D screen anymore. However, recent studies have made first attempts at a solution to this problem (24). These differences to conventional eye tracking studies on 2D screens should be considered when planning the data analysis.

## Pilot Study as an Example Application

In the following section, we present procedures and data that we collected during a pilot study in our virtual city. It is a follow-up of a study conducted in our lab investigating coding of spatial information about houses and streets in the real world (25). In the respective study, subjects performed various tasks involving photos of houses and streets of their hometown, i.e., Osnabrück, as stimuli. However, the familiarity of the subjects with the stimuli could only be assessed subjectively afterwards. Therefore, we piloted a second round of experiments designing a virtual city and including a 30-minute exploration of it. In this manner, we could control and monitor the exploration behavior of the subjects and objectively assess their familiarity with the different stimuli. In the presented pilot study, we focused on the subject’s visual behavior while exploring the city in VR. Investigating where subjects look while they explore a virtual city broadens the scope of data available for understanding their navigational behavior. As spatial knowledge is supposed to improve with higher familiarity, getting a more objective measure about subjects’ familiarity with the test stimuli is of great importance. The following full study is ongoing and will be reported separately.

### The Pilot Study

The reported study investigating spatial knowledge acquisition contained a 30-minute training session in Virtual Reality during which the participants were freely exploring the city called Seahaven. This virtual city was designed in a way so that it would be optimal for the tasks of the original study (25). This means that it does not include high landmarks and is not structured in a regular grid, i.e. Manhattan style. Furthermore, the houses display a wide variety of styles and looks to be easily distinguishable from each other because they serve as stimuli in the post training tasks of the full study. Overall, the city consists of 214 houses distributed in a sophisticated city layout. The VR environment also includes a moving sun to provide natural lighting conditions and the means to estimate cardinal directions.

In our pilot study, we recorded data of 31 subjects, two of which experienced motion sickness and could not finish the session. During two sessions, technical difficulties led to incomplete datasets. Thus, the data from the remaining 27 subjects (11 female, 16 male) with an average age of 24.2 is presented here. Since these were the pilot measurements, we changed minor aspects of the 3D environment during measurements of the first 10 subjects to improve the design. This included adding missing colliders, fixing misplaced objects or disjoint sidewalks, rescaling houses that looked too big/small, rescaling the sun and the player figure. Due to these changes, we will not present our data as actual results on spatial navigation. Nevertheless, due to the minor nature of these changes, we do not expect any relevant influence on the results. In this paper, we instead use our data as examples of an experiment in VR and to demonstrate the use of eye tracking data. We aim at illustrating the methods by applying them to one specific experimental setup.

**Figure 4.**
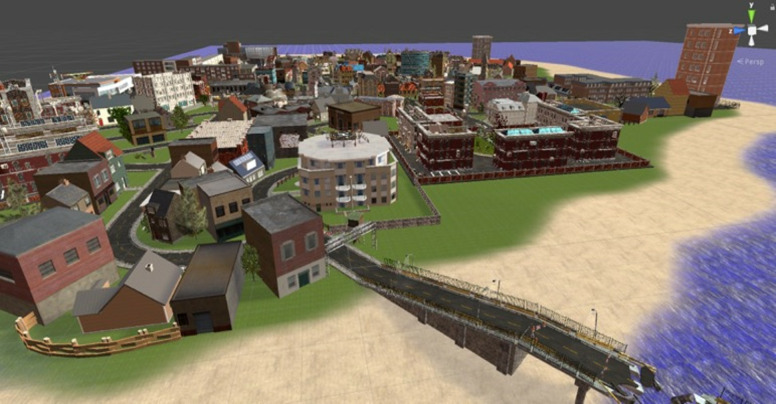
Seahaven - Surrounded by an ocean, the subject is forced to stay in the city. Many different houses and streets can be explored.

### The Structure of Experimental Sessions

**Figure 5.**
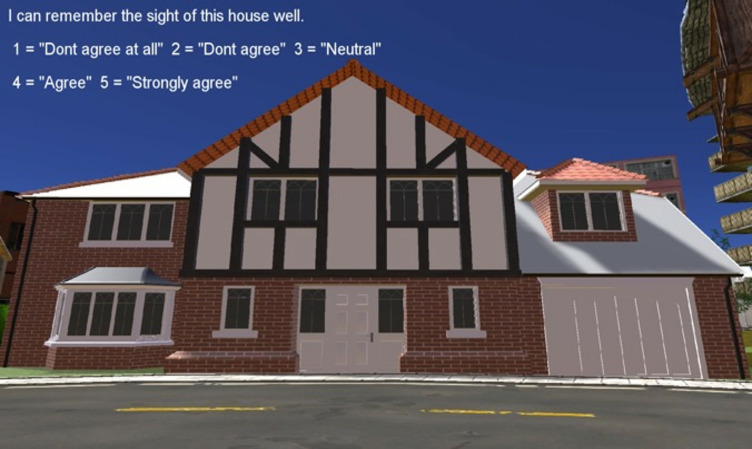
Design of the task. Two statements had to be rated for each picture. Here the familiarity rating is shown. After the response the same picture is shown again with the navigation question.

Before the start of the session, subjects receive an introduction into the study and give written consent. After explaining the mechanisms of VR and the potential risk of motion sickness, the headset is placed on the subject’s head, the interpupillary distance is adjusted by the subject, the eye tracker is calibrated, and the subject becomes accustomed to movement in VR inside of the small practice area. Once the subject felt comfortable, he or she was positioned in the VR city Seahaven and spent 30 minutes of free exploration inside the town. At the end of the session, the subject subjectively estimated north by turning into that direction. Then the session was terminated. A short task followed in which we presented to the subject pictures of 50 houses that were located inside the VR city. Those houses were randomly chosen and evenly distributed over the city. All subjects saw the same 50 houses in random order and had to rate two statements for each image (Figure 5). The first statement was “I can remember the sight of this house well”, to which we referred as the familiarity rating. The second statement was “I am confident that I could find my way back to this house”, to which we referred to as the navigation rating. The ratings ranged on a Likert scale from one, representing “Don’t agree at all,” to five, representing “Strongly agree.” There was no time constraint for giving an answer. After the task, the subject filled out a short questionnaire about its experience in VR. As an incentive, subjects were either given a monetary compensation or one credit needed for the study program. Each session lasted about one hour.

### The Setup

For our work, we decided to use an HTC Vive Headset with two base stations for motion tracking and one controller for movement in the virtual world (Figure 1A, B). Our eye-tracker is the HMD eye-tracker by Pupil Labs (Figure 1C). The eye-tracker is specifically designed for the HTC Vive and can easily be installed in the head mounted display. We send messages from Unity to Pupil Service via the IPC backbone. Code of our solution to communicate between Pupil Capture and Unity can be found in our GitHub repository^16^.

**Figure 6.**
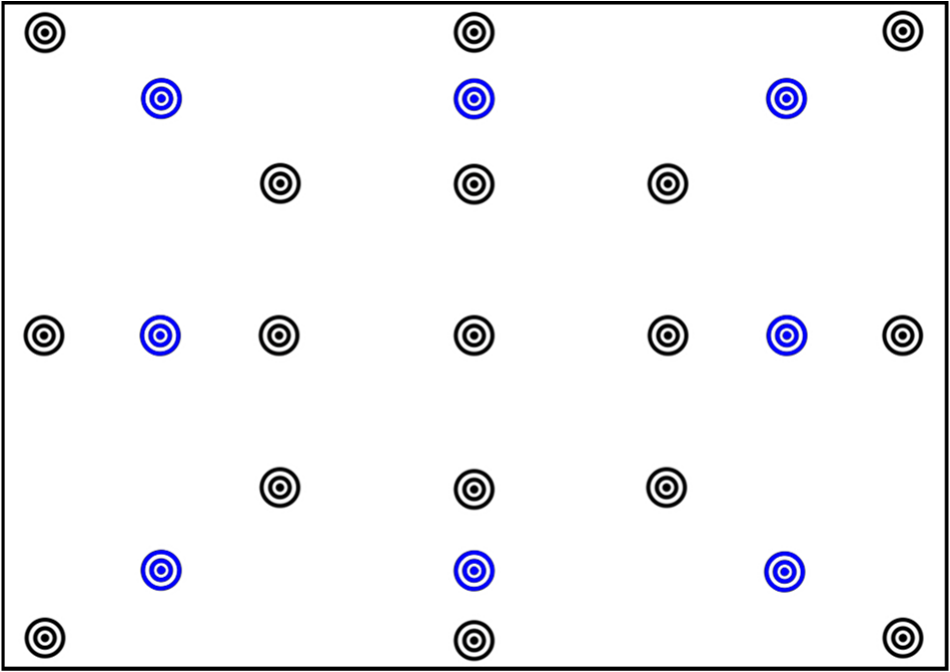
Points used for Calibration (black) and Validation (blue). The fixation point is shown in one of the locations at a time.

Figure 6 shows the distribution of the calibration and validation points we use for our studies. To secure that the eye tracker has a good accuracy over the whole visual field, i.e. also in the area between calibration points, we showed the fixation points during calibration at different locations than the ones during validation. With these measures, it is possible to routinely achieve a precision of the eye-tracker of 1.5° or better. We only used overall averages for the validations during the experiments and to decide whether a subject is calibrated well enough (if the average accuracy is below 2° no measurement can be performed). Post-hoc analyses show that the accuracy on the center point is on average higher than on the points in the periphery. However, the validation average accuracy excluding the center point usually still stays below 1.5 degree.

For software development we use Unity. The data analysis is done in Unity and MATLAB.

### Units in Seahaven

**Figure 7.**
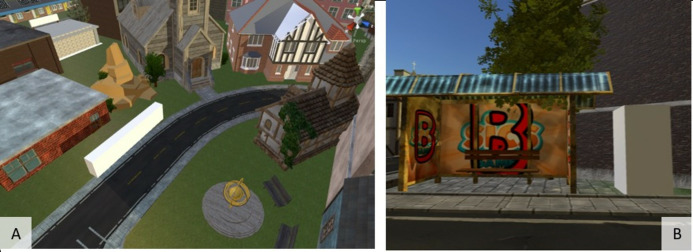
Size relations in Unity. (A) 10x2x1 Units cube. (B) 1x2x1 Units cube standing next to a bus stop. This is also the size of the player.

It is important to scale all objects of the virtual city in an intuitive way so that the subject gets a natural impression of the virtual city he or she is exploring. As Seahaven includes various types of houses from different sources that differ in style and size, we made an effort to scale all models to achieve natural proportions. We sized everything in Seahaven so that approximately one meter distance in the real world resembles one Unity unit. Figure 7 shows blocks of standard size to give an intuition of the scaling. The Player figure, which was not visible to the player, was approximately two meters high (Figure 7B). The approximate correspondence of one unity to one meter can later be used to interpret the results.

### Recording of Basic Variables

To extract information about a subject’s visual behavior, it is necessary to check basic variables at predefined intervals. We put the data extraction into a loop with a fixed frame rate and additionally save the data together with time stamps. At this fixed frame rate, we can then check for certain information and save it, such as the gaze direction and the player’s position.

For an objective assessment of familiarity, we collected information on the time each participant viewed each house while exploring the virtual city. We were interested in the specific house that was looked at and the distance from which it was looked at to correlate this with the performance in later tasks. We repeatedly checked this information 30 times a second and stored the results for later analysis. With the same method, we also saved other information about the subject such as head orientation and position.

### Measurement and Visualization of Exploration Behavior

**Figure 8.**
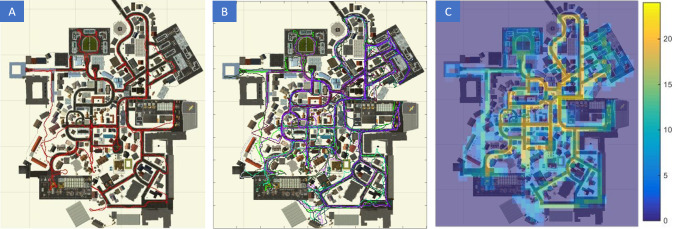
(A) Map of a single subject's walking path during a 30-minute session. (B) Map with walking path of 27 subjects. (C) Number of subjects that visited a certain area of the city.

First of all, we were interested to see where our subjects were during their free exploration of the virtual city. Therefore, we visualized our subjects’ navigation behavior by displaying its walking path on a map. In figure 8A, we show the path traversed by a single subject within a 30-minute session. Figure 8B displays the walking paths of all subjects that demonstrates an even coverage of the whole city. Figure 8C shows how many subjects were in the different areas of the city, which also reveals an even coverage of Seahaven. Therefore, we can conclude that within 30 minutes of free exploration, most of the virtual city was visited.

As a second step, we wanted to evaluate how many houses were looked at during the exploration time. The recorded eye-tracking data showed that during a 30-minute session on average 177.9 houses were seen, which corresponds to 83% of the city. A specific house was on average looked at by 22.5 out of 27 participants. 97% of the houses were seen by more than half of the subjects. In conclusion, our results suggest an even distribution of houses seen among the subjects.

**Figure 9.**
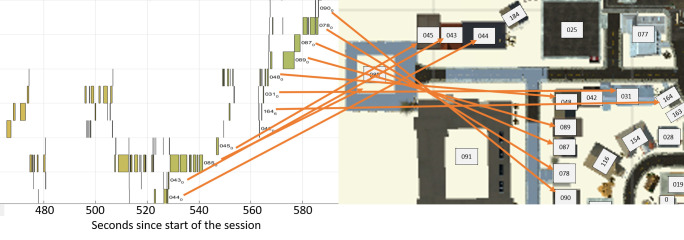
Excerpt out of a timeline of a full session. Represents houses on the x-Axis (Names are numbers between 001 and 200 + a rotation of 0, 3 or 6). Yellow blocks represent timespans during which a certain house was looked at. Orange arrows point to the house on the map.

To calculate the familiarity with a single house, we investigated how long a house was looked at. The results revealed that a single house was looked at for 5.53 s on average. An investigation of the most and least viewed houses (overall dwelling time) revealed that bigger house complexes and more extraordinary, free-standing houses were looked at longer than ordinary houses placed in a row along a street. By extracting the timestamps of each recording, we obtained a timeline representing which object was seen at which point in time. Figure 9 shows an excerpt of one subject’s timeline with stacked horizontal timelines for each house. The yellow blocks represent times in which the respective house was looked at. Houses were usually looked at for a certain time interval with some short jumps to other houses in between. As a familiarity measure we used the time that a single house was looked at extracted from the timeline of viewed houses.

**Figure 10.**
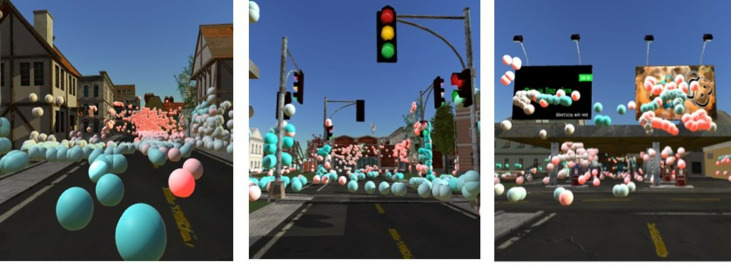
Visualization of gaze points from one subject. Spheres represent hit points of the gaze vector. Color-coded by distance from which it was looked at during the session (far->close = red->blue).

Next, we wanted to visualize subjects’ viewing behavior inside of the 3D environment (Figure 10). We calculated the gaze vector using the ray casting technique and marked each hit point in the virtual environment with a colored sphere. During this procedure the information on which objects were looked at were simultaneously extracted. For better visualization, we color coded the spheres by the distance from which the subject had looked at this specific point (far->close = red->blue). It is important to note, that the images shown in figure 10 do not correspond to the subject’s point of view from which he or she looked at the marked spheres. This means, that if you for example see many different colored spheres on the same object like in figure 10 on the right, the subject looked at this object from a far distance and then came closer to look at it (or vice versa). Since the spheres are visualized in a 3D model we can now walk around in this model and explore the subject’s gazes. Because we only use few box colliders for each house to improve the performance of our application, some hit points do not exactly correspond to the object’s shape. For example, this can be seen in the empty space between the two ad panels in the picture on the right. Nevertheless, directly marking the visual hit points in the 3D environment offers a convenient and flexible way to visualize and investigate the viewing behavior of subjects exploring the virtual city Seahaven.

### Analyzing the Eye Tracking Data in Relation to Other Variables

The advantage of eye tracking in VR is apparent once you start analyzing your eye tracking data in relation to other data recorded during an experiment. Such data may be the subject’s position or rotation, walking speed, actions in the VR environment or performance in a task inside or outside of VR or subjective evaluations. In the following section, we will present a broad array of such analyses using our study as an example.

### Correlation of Viewed Houses and Task Performance

**Figure 11.**
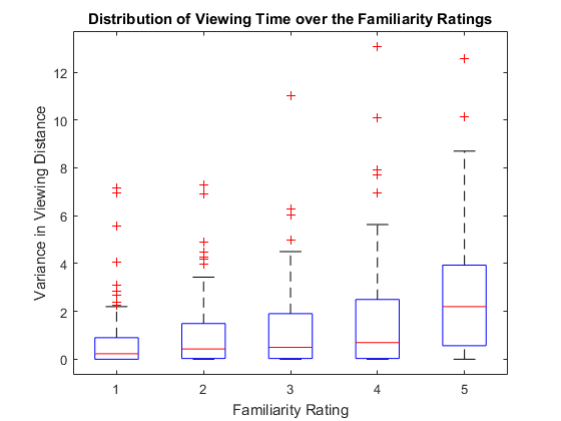
Houses that were looked at for a longer time had an overall higher familiarity (and navigation, not depicted) rating.

The previous study (25) assessed the familiarity of the stimuli, i.e. houses of Osnabrück, by a subjective familiarity rating after performing spatial tasks. However, it was not possible to objectively assess how often subjects actually visited the respective locations. In VR, we can extract the objective measure of the total time spent looking at a specific house as an objective familiarity measure and relate this to a subjective measure of familiarity. Therefore, subjects had to answer a familiarity question in our pilot study ("I can remember the sight of this house well."). The answers ranged on a scale from one to five with one representing "don't agree at all" and five representing "strongly agree". The average rating over all subjects was 2.81. The correlation between viewing duration and subjective familiarity rating was 0.293. With the same procedure, we calculated the averages for the responses to a question regarding spatial navigation ("I am confident that I could find back to this house."). Here, the subjects' overall average was at 2.46. Between the navigation rating and viewing duration the correlation was 0.316. Figure 11 shows the distribution of viewing time per house and subject respective to the familiarity rating given to it. The overall results support our expectation that the subjective familiarity rating of a house increases with increasing amount of time a house was looked at. However, the correlation was lower than we expected.

**Figure 12.**
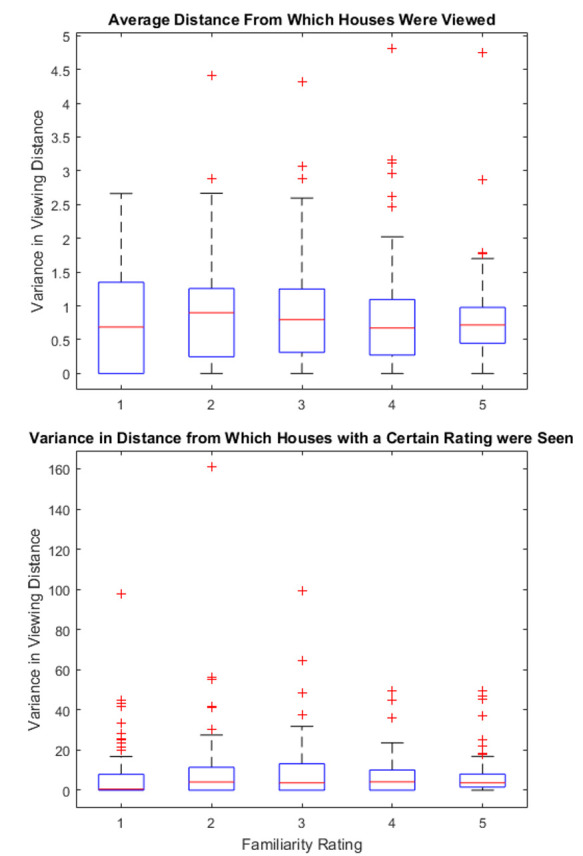
(Top) Average distance from which a house was seen. (Bottom) Average variance in distances.

Next, we investigated whether the distance from which a house was seen had any influence on how familiar the subjects would rate it (Figure 12). We could not find a strong relation between familiarity and viewing distance of a house (Correlation of -0.047). Also, for the navigation rating no correlation with the average viewing distance could be found (Correlation of -0.056). The variance in distance from which a house was seen also did not seem to affect the ratings.

### Spatial Distribution of Viewing Points

The VR setup allows determining the full 3D distribution of viewing points^17^. In most laboratory experiments 2D visual stimuli or projection of the fixation location onto a 2D plane is used. Instead in VR, we have the true 3D information available and can construct a 3D distribution of viewing points using 3D heat maps to visualize gaze movements. The generation of 2D heat maps after collecting eye-tracking data to determine spatial bias is by now common practice. Using VR, we can now also look at the bias in 3D space adding distance to the 2D plane. The distance from which subjects looked at objects will be our third dimension for a 3D heat map and can give further insights into the visual coverage of a 3D environment.

**Figure 13.**
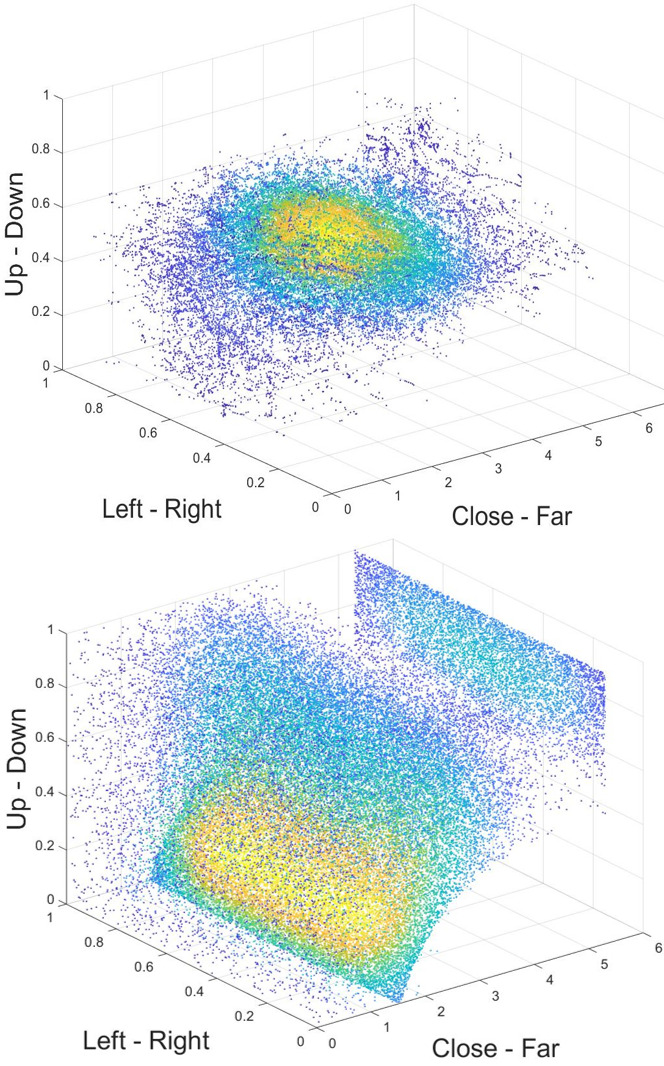
3D Heat map of one subject. Points are colored according to the number of close neighbors of a point and thereby code the density of points in this area (yellow=high density, blue=low density). Close-Far axis shows Log(Distance). (Top) Original gazes show concentration at center of visual field at a medium distance. Maximum density at distance of ln(23)=3.1. (Bottom) Randomly distributed gaze vectors in x and y plane show the intrinsic properties of the VR environment.

An example of a 3D spatial distribution for a single subject is shown in figure 13. To have increased resolution at small distances, we plotted the distribution of gaze points as a function of the natural logarithm of the distance. A distance value of around ln(160) ≈ 5.0 represents the far clipping plane in our virtual world, which is the furthest distance the subject can see. The plane of points displayed slightly behind the clipping plane represents gazes that did not hit any object, for example, gazes into the sky. The figure shows that the subject looked most often straight ahead at a medium distance of about 23 units (~23 meters).

**Figure 14.**
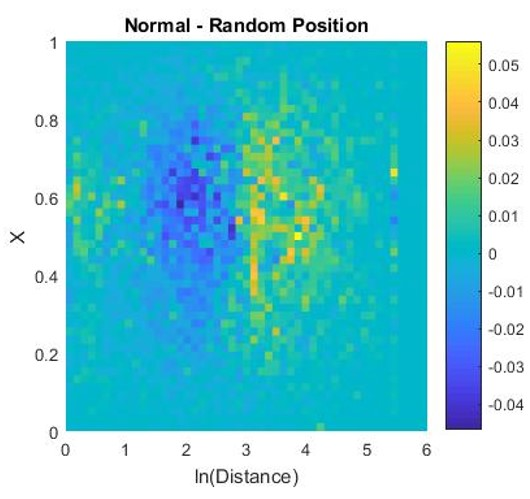
Normal heat map - heat map of hit points resulting from shuffled gaze vectors (One subject). It shows a systematic bias for bigger distances.

This 3D heat map includes properties of the viewer as well as of the environment. Specifically, the statistics of distances between user and houses might play a role. To adjust for the properties of the environment, we used two different randomization methods. First, we shuffled the original gaze vectors and randomly assigned them to the recorded positions of the user. This means that a gaze vector from the beginning of the session could be used at a position (in head centered coordinates) visited towards the end of a session and hit a different object at a different distance. The resulting heat map looks similar to the original heat map with a maximum density at ln(14)=2.6. On the horizontal and vertical dimension, the data points are the same since we used the original gaze vectors from this subject. The shuffling assigns individual points new values on the distance axis. When subtracting the randomized data from the original heat map, we can see that there is a higher density of points at further distances for the original hit points than for the hit points resulting of the shuffled gaze (Figure 14). This indicates that subjects systematically looked at objects that are further away. Please note that this is the opposite to results obtained under classic lab conditions with static images displayed on 3D monitors (21). This stresses the importance of investigating behavior under natural conditions.

In the second control, we randomly created new gaze vectors with a flat probability function and applied them to the recorded positions of the subject. When you look at the distance axis, you see a curve shaped pattern and a plane at the furthest distance (Figure 13, bottom). The accumulation of points at the far clipping plane represents all gazes that go into the sky or so far into the distance that objects were no longer featured on the display anymore. The curve shaped pattern visualizes gazes that hit the ground. The further down the gaze goes, the earlier it hits the ground and is therefore shown at a shorter distance in the heat map. It looks very different to the heat maps created from original gaze vectors and reflects properties of the 3D world much more. This indicates that the original heat map without any randomization displays the actual viewing behavior and is not just a product of its environment.

### 2D Gaze Visualization and Analysis

**Figure 15.**
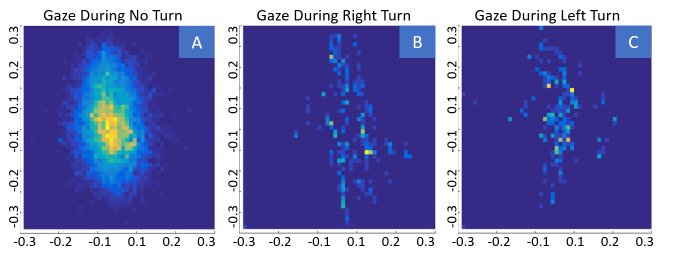
Sample Data of two subjects. (A) Heat map of gazes while the subject is not turning. (B) Gazes in 10 frame time windows while the subject is making a right turn >20 degree. (C) Gazes in 10 frame time windows while the subject is making a left turn >20 degree.

One question that arose during observation of many VR sessions is if the gaze of a subject is different when he or she turns the head. There are various ways to look at the 2D gaze of a subject with regard to other parameters of interest. In our case, we were interested in eye movements, which are correlated with different body movements in the virtual environment. For this, we split the recorded eye positions into three groups: Eye positions in a 10-frame time window around a right turn of more than 20 degrees, eye positions in a 10-frame time window around a left turn of more than 20 degrees, and eye positions during all other frames where the subjects’ rotation did not change by more than 20 degrees. When plotting these three classes, we saw that left and right turns were often accompanied by gazes in the respective direction (Figure 15). Thus, for these large turning angles of the head or body synergistic explorative eye movements dominate over compensatory stabilizing eye movements (26).

**Figure 16.**
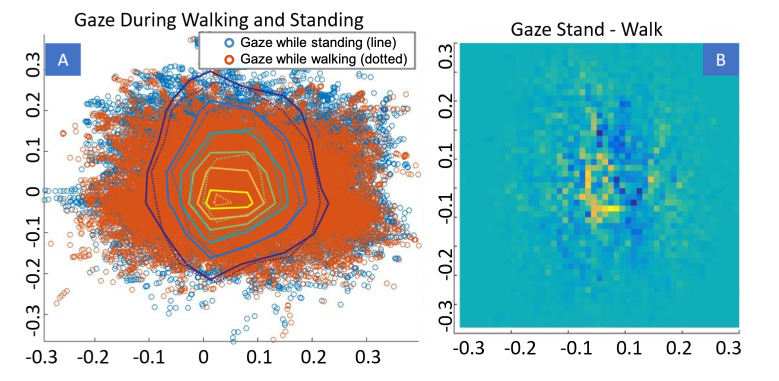
Gaze during walking compared to gaze during standing. (A) Scatterplot of the two gaze classes (Orange - walking, Blue - standing). (B) Heat map of (gazes while standing) – (gazes while walking).

Additionally, we examined eye movements during walking compared to eye movements while standing (Figure 16A, B). Figure 16A demonstrates that gazes while standing (orange) have a higher concentration in the center than gazes while walking. The same can be seen, when subtracting gazes while walking from gazes while standing in a heat map (Figure 16B). This indicates that under the given experimental conditions, there is more intense visual exploration of the environment while the subject is walking. However, this could also be a side effect resulting from the way subjects navigate in the virtual world. Since the walking direction of the subject is determined by the orientation of the headset, head movements during walking make it harder to walk in the desired direction. This could make the subjects resort to the use of more eye movements to the periphery instead of moving the head while they are walking. While standing, the subject can simply move the head around and look more at the center of the HMD screen.

## Discussion

In this paper, we have described and discussed a method to easily track eye movements inside of a virtual environment. This is done by using basic geometric calculations i.e., first constructing a 3D gaze vector out of the 2D pupil position and then intersecting this vector with objects in the 3D world. We’ve also made suggestions for analysis that can be applied to the recorded data as well as some details of the technical implementation and possible sources of problems. We will now progress with some more in-depth discussion of this technology, in particular the limitations and possibilities created by it.

### Limitations and Possibilities of VR

During the development of the VR city and the following pilot study, we came to the conclusion that Virtual Reality is a very powerful tool for conducting research. It provides a large amount of valuable data while resembling the real world well and giving the subject the possibility to move relatively freely. A virtual environment is closer to a natural condition than the usual lab environment and is a more controlled condition than a study conducted in the real world. This makes it a highly valuable tool to explore a wide range of questions in the area of spatial navigation and many other fields. The option to track a subject’s body movements and eye movements with a reasonable precision gives the researcher a considerable amount of information on the subjects' behavior in the virtual environment. It makes it possible to analyze subjects’ behavior in relation to what they looked at as well as analyzing where subjects looked in relation to their behavior. The possibility to investigate the interaction of body movements and gaze movements in such a precise and simple manner opens possibilities to answer many new questions. Also, the creation of 3D heat maps serves as a demonstration of this valuable tool for investigating viewing behaviors. The possibility to visualize gaze patterns inside of the 3D model can also be a useful tool when presenting research or constructing new hypotheses. There are many more ways to use the combination of VR and eye tracking to your advantage. We see much potential in this new technology and many opportunities for it to foster future research.

However, these advantages come with setbacks. The main problem is currently the tendency for people to experience motion sickness in VR. In our study 12 out of the total of 31 subjects reported in a questionnaire that they felt mild discomfort due to motion sickness during the session. Additionally, two participants had to interrupt the training due to this reason. Only 6 of our participants indicated in a questionnaire that they usually get motion sick in cars or other vehicles. This high rate of motion sickness among subjects made it difficult to conduct any experiments lasting longer than 30 minutes and lead to a number of participants having to stop during a session. Motion sickness also prevents the subjects from being fully involved in exploration (or any other task) since their focus will center on controlling their dizziness or nausea. Additionally, motion sickness leads to an adapted navigation behavior, which means for example, slower walking, not turning the head while walking, stopping to look around or looking only at one object for an extended amount of time to combat the motion sickness. All these adaptations of subjects who experience motion sickness hinder our ability to analyze their natural navigation behavior.

One potential solution to this problem could be to split the session into shorter time spans. For example, three sessions of 10 minutes with two breaks in between instead of one session of 30 minutes. Another way to cope with this is to find a more natural way of moving through the virtual environment. This would solve two problems at once. It would reduce motion sickness and make the simulated conditions more closely resemble natural conditions. To have continuous movements when exploring our virtual city, the subjects are sitting on a rotatable chair and only influence their movements by operating the controller with their thumb and rotating the chair. This does not resemble natural movement and it is unclear in how far it influences navigation behavior. For example, it makes it possible to walk sideways and backwards, which is an unusual occurring phenomenon among people. Also, the walking direction is influenced by the orientation of the head and moving in one direction while looking in another requires some training and coordination. However, presently it is hard to find a more natural way to move in VR, which does not cause motion sickness. Solutions used in games like teleportation are not feasible for many research questions.

Something that could also help is to give the subject an active task, which distracts from motion sickness. This was the suggestion of a couple of subjects who reported that without a task they had a lot of time to pay attention to how they feel and noticed the motion sickness easier than they would expect when having a task. However, finding a good task, which does not interfere with the main purpose of an experiment is difficult. Since every task would influence the subject's navigation behavior, it is problematic to add this into the session's setup. On the other hand, natural navigation behavior is usually driven by a task or a goal, so by accounting for the influence of a well-designed task in the analysis, this could be a possible solution as well.

Overall, these drawbacks should be considered when designing an experiment in VR. Some research questions might have to be reformulated or answered using a different method. However, there remain many applications where VR is a powerful tool to collect information about various topics that were previously extremely difficult to assess. With the pace of current developments in the field of VR, increasing computational power and more efficient algorithms, we expect solutions for many of these problems to come up in the close future.

### The Use of Eye Tracking in VR

Eye tracking in VR is a good solution combining ad-vantages of classic laboratory setups with fixed monitor screens and the real world. This paper presents you with a method to implement eye tracking into any VR set up, with a method of defining objects of interest and to collect data about the time at which an object was looked at as well as the distance of the observer to the object and the exact point where he looked. With the 3D visualization, you can get an impression of which objects and which parts of an object were looked at the most.

Of course, there are also some minor drawbacks. One drawback is that subjects’ wearing glasses cannot always participate in the VR experiment. Another factor is that the eye tracker needs to be calibrated and validated which requires additional time and can prove to be disruptive during a longer session. Also, the participant cannot move the headset on the head anymore after calibration which is why you need to make very sure that it is sitting on the face comfortably in the beginning of the session. Fast movements in VR could lead to the headset moving on the face and should be avoided. During analysis, it can be a problem that some eyes are tracked more easily than others (usually bright eyes are better than dark eyes). Since we only log gaze points with a confidence above 0.5 some subjects have many more gaze points to analyze than others.

### Comparability of Behavior in Virtual Reality to Real World Behavior

After considering all the above-mentioned factors, one question is left: How much of the behavior that we observe in virtual reality corresponds to actual behavior in the real world? In other words, can we generalize our results acquired in VR as normal human (viewing) behavior? The improvement of real-world resemblance compared to experiments on a normal screen, often with head fixed conditions, seems to justify the use of VR. However, there are a couple of differences that we need to keep in mind when interpreting our results.

First, the screens have a higher effective resolution in the center compared to the outer parts (20). This influences the relation between head and eye movements since the subject needs to move the head to see an object of interest at a high resolution in VR. The rather limited field of view of current HMDs could also contribute to this effect.

Concerning viewing behavior, one of the main differences between real and virtual world is the vergence-accommodation-conflict. Since the two screens always have the same distance to the eyes, our lenses do not need to shift focus to look at objects at different depths. Since all information is given on a single focal plane instead of arriving from many focal planes as in the real world, there is also less depth information conveyed. The lack of depth of field cues like blur lead to a different perception of the size of objects (16). There are currently several solutions to this problem being developed in VR including light field displays (27) or blur of the periphery by using eye tracking ( 18, 28). However, we have to consider, that similar limitations apply to typical monitor setups, i.e. it is not a VR specific problem. Still, the different presentation of the virtual world to the subject should be considered since it could influence visual behavior.

Natural walking within a bigger virtual environment is hard to accomplish since it would require omnidirectional treadmills, which are being developed^18^, but today remain still difficult and expensive to acquire on the consumer market. Another solution could be to install a tracking area as big as the virtual environment, but this requires much space and a solution for cables like a VR backpack^19^ or wireless information transmission^20^. There is a lot of development going on in these areas, which is why we expect new and affordable solutions coming up in the near future.

Experiments which involve interaction with objects might require a hand tracking system instead of the use of controllers. There are some solutions available involving hands tracking with infrared cameras^21^ or systems using gloves^22^, which can also provide some force feedback^23^.

Whether these differences to the real world influence the results, depend very much on the research question, but all of them should be considered when designing the experiment and assessing the results.

### Conclusion

Overall, eye-tracking is a highly useful tool to investigate various questions in VR. It works precisely and with the method presented in this paper, you can easily match the eye gaze with the different objects in a virtual environment. The possibility to model various environments and control every aspect of it is highly valuable in research and should be further exploited in the future. On the downside, VR poses some challenges to the experimenter concerning natural movement and motion sickness to which a perfect solution has yet to be found. Although due to the rapid changes in the field of VR technologies we anticipate that solutions to these problems will be discovered soon. In the end, the possibilities outweighed the disadvantages of the use of VR for our research and it opened a lot of new opportunities to analyze our subjects’ behavior in the VR city. In conclusion, we believe that eye tracking in VR has enormous potential for research and can be used to great effect in answering further questions about human cognition and behavior. We hope to have sparked some new ideas with this paper and that the advantages of this technology will be put to further use in the future.

## Ethics and Conflict of Interest

The author(s) declare(s) that the contents of the article are in agreement with the ethics described in http://biblio.unibe.ch/portale/elibrary/BOP/jemr/ethics.html and that there is no conflict of interest regarding the publication of this paper.

## Acknowledgements

We are happy to acknowledge technical support by Petr Legkov. We gratefully acknowledge support by “VRFlow Suite: Embodied Engineering in der Produktionstechnik“, EFRE ZW 3-85011202 as well as support by Deutsche Forschungsgemeinschaft (DFG) and Open Access Publishing Fund of Osnabrück University.

## Footnotes

https://www.oculus.com/rift/

https://www.vive.com/eu/product/

https://www.playstation.com/de-de/explore/playstation-vr/

https://www.microsoft.com/en-us/store/b/virtualreality

https://vr.google.com/cardboard/

This may be different by the time you read this paper.

https://unity3d.com/

https://www.unrealengine.com/

http://www.worldviz.com/vizard-virtual-reality-software/

https://pupil-labs.com/vr-ar/

https://www.tobii.com/tech/products/vr/

https://www.getfove.com/

https://www.displaylink.com/vr

In most implementations of teleportation, the player points to the desired location with help of a virtual laser beam. The screen fades to black and after a short delay the virtual world fades in again with the player appearing at the desired location.

A child object inside of Unity is an object that is subordinate to another object and moves respective to its parent object.

https://github.com/vkakerbeck/NBP-VR-Lab

Since we do not work with fixations here and only look at the subjects’ gaze 30 times a section we will call the hit points of these gazes viewing points.

http://omnifinity.se/ , http://www.virtuix.com/, https://www.cyberith.com/

http://vr.msi.com/Backpacks/vrone

https://www.tpcastvr.com/

https://www.leapmotion.com/

https://hi5vrglove.com/

https://vrgluv.com/
